# The Order of Draw during Blood Collection: A Systematic Literature Review

**DOI:** 10.3390/ijerph18041568

**Published:** 2021-02-07

**Authors:** Giacomo Bazzano, Alessandro Galazzi, Gian Domenico Giusti, Mauro Panigada, Dario Laquintana

**Affiliations:** 1 Direction of Healthcare Professions, Foundation IRCCS Ca’ Granda Ospedale Maggiore Policlinico, Via Francesco Sforza 35, 20122 Milan, Italy; giacomo.bazzano96@gmail.com (G.B.); dario.laquintana@policlinico.mi.it (D.L.); 2 Corso di Laurea in Infermieristica, Dipartimento di Medicina Sperimentale, Università degli Studi di Perugia, Piazzale Gambuli, 06129 Perugia, Italy; giandomenico.giusti@unipg.it; 3 Department of Anesthesia, Intensive Care and Emergency, Foundation IRCCS Ca’ Granda Ospedale Maggiore Policlinico, Via Francesco Sforza 35, 20122 Milano, Italy; mauro.panigada@policlinico.mi.it

**Keywords:** blood collection, blood collection tube, nursing, order of draw, phlebotomy, preanalitical phase, venipuncture

## Abstract

Blood collection is one of the most common nursing procedures and is not devoid of complications. The order of draw during blood collection is a controversial theme. We aimed to define the efficacy of the order of draw during blood collection to guarantee an exact biochemical result. We carried out a systematic literature review on PubMed, Scopus, Web of Science, CINAHL, Embase, Joanna Briggs Institute, Cochrane Library, and Google Scholar. Articles written in English and published from 2000 to 2020 were considered suitable. The analysis of the 11 articles included highlighted different opinions; however, the most recent evidence declares that the cross-contamination caused by the incorrect order of draw is a trait only in the open system of drawing. The most recent evidence affirms the negligible effect of the order of draw during blood collection when using the closed blood collection system, while it is recommended when using the open collection system.

## 1. Introduction

One of the most common nursing procedures is blood collection. Indeed, this is a technique practiced in all departments that allows investigation of the patient’s blood values, but it is not without risks [1]. Therefore, it is extremely important that the results obtained are true and without mistakes. It is well known that the pre-analytical phase is essential for good-quality laboratory results; several studies have shown that almost 75% of all analytical mistakes occur during blood collection [2,3]. The types of errors that could occur in the preanalytical phase range from wrong patient identification to filling an incorrect sample tube and other mistakes in the course of the procedure [2].

The World Health Organization and the Clinical and Laboratory Standard Institute have edited specific guidelines to ensure the good quality of the preanalytical phase [4,5]. However, in the biomedical literature, there are different opinions about one special item of the recommendations: the order of draw of blood collection tubes. This particular finding has been the focus of numerous studies since 1977, when Sun and Knauf reported a case of spurious hyperkalemia and hypocalcemia due to suspected blood contamination [6]. But it was after the publication of the study by Calam and Cooper [7], in 1982, that the Clinical and Laboratory Standard Institute began to modify the recommendations slightly throughout the years, increasing the use of plastic instead of glass tubes and developing new additives [8]. Nowadays the order of blood draw established by the international guidelines is postulated to avoid cross-contamination between different additives contained in blood collection tubes, and it is as follows: blood culture bottle, non-additive tube, coagulation tube, clot activator, serum separator tube, sodium heparin, plasma separator tube, ethylenediaminetetraacetic acid (EDTA), blood tube, and oxalate/fluoride.

Additive contamination, when it occurs, does not only result in erroneous blood values but also in a waste of healthcare resources and, particularly, if not swiftly recognized, may negatively affect patient care [9,10]. In general, the carryover of additives could virtually increase or decrease some laboratory values. The contamination with EDTA can lead to spurious electrolyte results such as hyperkalemia, hypocalcemia, hypomagnesemia, hypozincemia, hypophosphatemia, and longer coagulation times. It can also modify other laboratory parameters like unsaturated iron binding capacity, bicarbonate, aspartate transaminase, alanine transaminase, lactate dehydrogenase, creatine kinase, alkaline phosphatase, and α-amylase [11]. Similar effects might be caused by citrate contamination [12]. The coagulation parameters could even potentially be altered by contamination with heparin [13]. Finally, hematological parameters could be modified by fluoride or oxalate because of their capacity to cause disruption to the cell membranes [9]. Despite this, several studies in the literature have shown deeply varying results and maintain a different viewpoints about the possibility of cross-contamination. The contamination is reported to occur through three different mechanisms. First, by direct transfer of blood from one tube to another; it is strongly recommended not to decant blood from one tube to another because of the contamination of blood due to the additive of the first tube. Second, by backflow of blood from the first tube into the second tube using the vacutainer system, the contaminated blood from the first tube could backflow into the needle and be transferred to the second tube. Third, by syringe needle contamination when pouring blood into different blood collection tubes, as blood is drawn with a syringe and tubes are filled one by one. If the needle of the syringe becomes contaminated by the additive of one tube, wrong blood values obtained from the subsequent tubes could occur [14,15].

The differences between closed and open blood collection are multiple. The blood collection system is “closed” when the blood drawn passes directly into the tube with no exposure to the environment or to healthcare professionals. The most used closed blood collection system is the winged butterfly needle and luer-lock connectors with vacuum blood collection tubes. This system makes it easier to take multiple samples from a single venipuncture [4]. The blood collection system is called “open” when, during the procedure, the blood is exposed to the environment and to healthcare professionals. The typical open blood collection system is a syringe combined with a needle [4]. The latest studies have looked at whether the order of draw remains a problem when using the closed collection system, modern materials, and modern techniques, or whether it is simply a relic of the past. Nowadays the order of draw using the closed blood collection system, however, continues to be recommended based on common sense rather than on evidence [3,10,16].

The aim of this systematic review is to define the efficacy of the order of draw during blood collection to guarantee an accurate biochemical result.

## 2. Materials and Methods

### 2.1. Design

A systematic review of the international literature was carried out following the PRISMA methodology [17]. All types of articles (observational and experimental) in English were considered as potentially suitable. PubMed, Scopus, Web of Science, CINAHL, Embase, Cochrane Library, Joanna Briggs Institute, and Google Scholar were consulted. The specific research question was formulated using the PICO (patients, intervention, comparison, outcome) model [17]. We considered as patients all humans subjected to blood specimen collection; as intervention, following the recommended order of filling tubes during venipuncture; as comparison, not following the recommended order of filling tubes; and as outcome, the accuracy of the biochemical results.

### 2.2. Search Strategy

The key terms used in the literature search included “order of draw” and “blood specimen collection” linked by Boolean operators. The search string was (“Blood Specimen Collection” [Mesh]) AND ((order of draw*) OR (order of fill*) OR (order of collect*)). We also applied the filter “Humans” during the literature search to minimize the number of articles not pertinent to the review. We also considered as eligible the articles found in the “Similar Articles” box and “References” during the main research. 

### 2.3. Inclusion and Exclusion Criteria

We included the studies that described the prevalence and effects of cross-contamination between different blood additives. Limitations concerning ethnicity, sex, co-morbidity, or other characteristics were not applied. Case studies of individual patients, letters, and editorials were not eligible. We considered only studies written in English and published from January 2000 to June 2020.

### 2.4. Search Outcomes

An evaluation process based on three levels was used: appropriateness of the title, evaluation of the abstract, and evaluation of the full text. Each evaluation level was analyzed separately by two authors, who examined all the bibliographic references judging whether they were potentially suitable. The results of each level were compared, and a third author resolved any disagreement.

### 2.5. Quality Appraisal

The quality of the approved studies was assessed using the Critical Appraisal Skill Program (CASP) checklist [18]. This checklist comprises 12 questions with a score 0 or 1 depending on whether the item is present in the article or not. The questions investigate the coherence of the study, the methods used, the precision of the results considering bias and confounding factors, the applicability to the population, and the implications for practice. We considered as suitable the articles with a CASP score equal to or higher than 9/12. The quality of each article was assessed by two authors independently. Any disagreement was dealt with effectively with the aid of a third author.

## 3. Results

### 3.1. Study Selection

**Figure 1:**
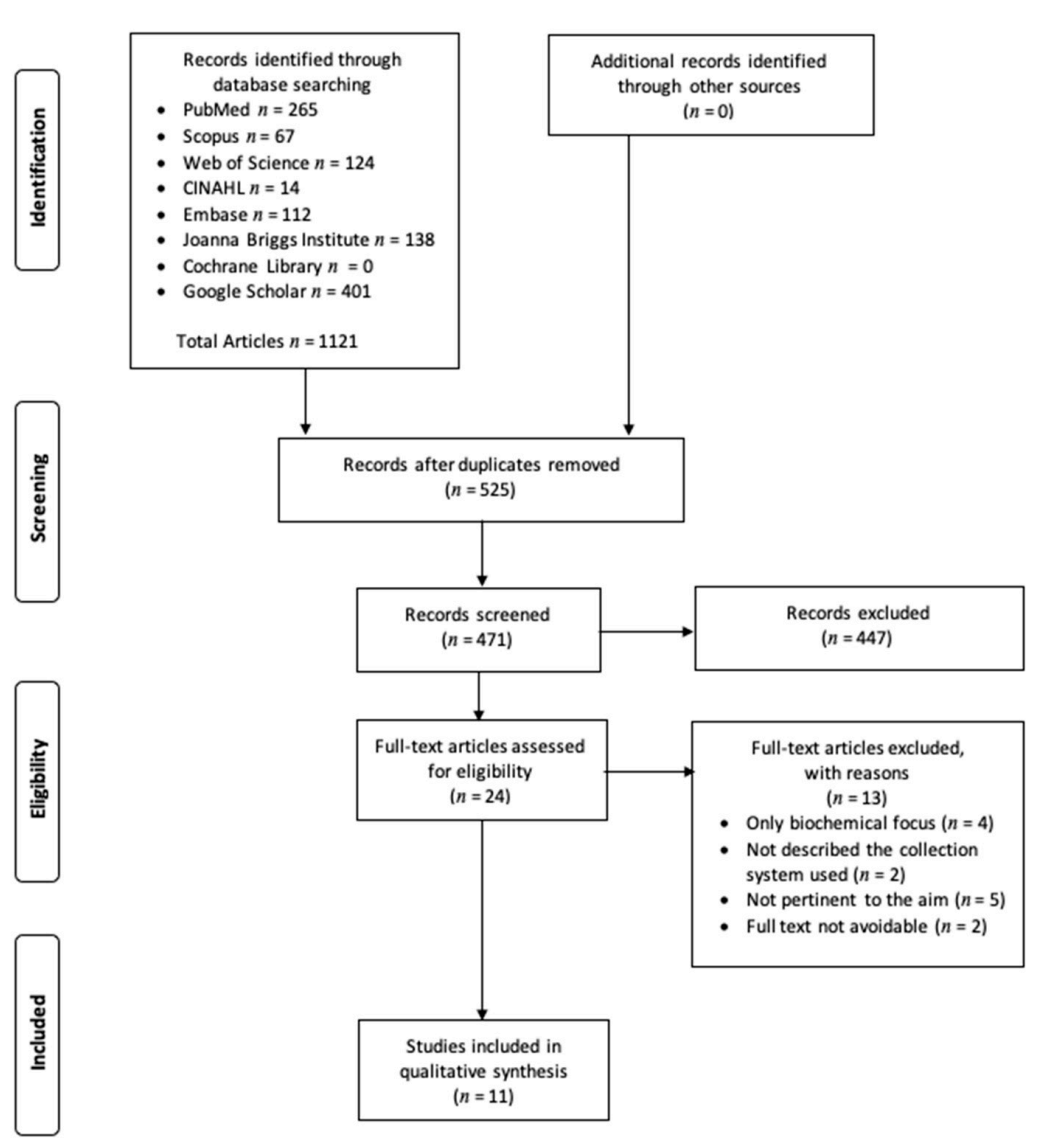
Search and selection flow diagram.

The initial search identified 1121 titles from PubMed (*n* = 265), Scopus (*n* = 67), Web of Science (*n* = 124), CINAHL (*n* = 14), Embase (*n* = 112), Joanna Briggs Institute (*n* = 138), Cochrane Library (*n* = 0), and Google Scholar (*n* = 401). Reasons for exclusion were not pertinent to the aim of the review, no description of the collection system used, only biochemical and not clinical focus, papers not written in English, papers written before 2000, no qualitative data was available, and full text was not available. After screening and deleting the duplicates, 11 articles were considered appropriate. Figure 1 shows the search strategy flow diagram used to obtain the results.

### 3.2. Study Characteristics

Of the 11 selected studies, 8 were observational, 1 in vitro, and 2 both observational and in vitro studies. The details extracted from the chosen studies are synthetized in Table 1.

We found various conclusions favorable and contrary to following the order of draw: 7 studies stated its negligible importance to prevent cross-contamination [13,14,19,20,21,22,23]; and 4 confirmed its necessity [15,24,25,26]. Considering all the observational and the two both observational and in vitro studies: 6 were in contrast with the international guidelines and 4 complied. The in vitro study confirmed that no contamination occurred during the procedure, so, in their opinion, the order of draw could not be followed. 

### 3.3. Efficacy of Order of Draw

The articles that sustained the importance of following the recommended order of draw were 3 observational studies and 1 in vitro and observational study. 

The prospective study of Cornes et al. [26] measured levels of serum EDTA, zinc, magnesium, calcium, and alkaline phosphatase in samples with serum potassium ≥ 6.0 mmol/L. A total of 28 of 117 hyperkalemic samples were contaminated with EDTA. The spurious values obtained were hyperkalemia, hypocalcemia, hypomagnesaemia, and hypozincemia. In conclusion they confirmed that EDTA contamination was common, and at low concentrations of EDTA, it can only be confidently detected by measurement of serum EDTA [26]. 

The evaluations asserted by Sharrat et al. [24] were announced after over a 1-month period of investigation of all the samples with more than 6.0 mmol/L of potassium or from patients with hypocalcemia, hypomagnesemia, and hypozincemia. During the period of the study, 12,895 samples were analyzed, and 289 presented hyperkalemia. Among these, 9 (3.1%) were identified as EDTA-contaminated by routine screening. Out of 7319, 569, and 295 samples processed for bone profile, magnesium, and calcium, respectively, 104 were hypomagnesemic, 133 hypocalcemic, and 139 hypozincemic. Among these samples, 22 were identified as EDTA-contaminated. Sharratt et al. concluded that factious hyperkalemia, hypocalcemia, and hypomagnesemia caused by EDTA contamination are relatively common [24]. 

The multicentric study guided by Cornes et al. [15] investigated the prevalence of spurious hyperkalemia due to EDTA contamination. In this work, the researchers analyzed the quantity of EDTA in the serum of patients with potassium level ≥ 6 mmol/L, and they stated that in 4.1% of all hyperkalemic samples, the abnormal values were due to cross-contamination between the EDTA-containing blood collection tube and the one containing heparin as anticoagulant. They concluded that hyperkalemia due to EDTA contamination is relatively common [15]. 

The last article according to the recommendation, by Fukugawa et al. [25], is about the contamination of thrombin in the blood collection tube containing sodium citrate for the study of the patient’s coagulation times. In these study, 100 paired blood specimens were collected in coagulation tubes before and after the serum tube. The parameters investigated were prothrombin time (PT), PT ratio, PT-international normalized ratio (INR), activated partial thromboplastin time (aPTT), fibrinogen, D-dimer, and fibrin monomer complex. The experiment revealed a minimal, without clinical relevance but statistically significant, effect of clot activators on the values of several coagulation parameters of the sodium citrate tube collected after the serum-containing tube. The PT, and consequently PT ratio and PT-INR, were shortened in the last blood collection tube in comparison with the first because of the presence of the clot activator. Nevertheless, the authors end their article maintaining that using the standard phlebotomy sequence, it may be acceptable to collect the coagulation tube after the serum tube [25].

### 3.4. Negligible Importance of the Order of Draw

Seven articles stated the negligible importance of the order of draw: 5 observational studies, 1 in vitro study, and 1 both in vitro and observational study. 

In the study conducted by Indevuyst et al. [20], 193 patients receiving oral anticoagulation were tested to investigate the importance of the order of filling blood tubes. The citrate-containing tubes were collected like the first tube, before and after an EDTA, heparin, or serum tube with clot activator. At the end of their study, no statistically significant influence on PT-INR and aPPT values was detected when blood was drawn after the heparin-containing tube. There was a contained, but statistically significant, bias on the aPTT when the citrate tube was collected as the first, after EDTA and after the clot-activator-containing tube. However, considering the small bias and the negligible effect on the patient’s clinical progression, the authors assert that the order of draw has no significant influence on clot values [20]. 

In their observational study, Cornes et al. [21] investigated the levels of serum potassium, calcium, magnesium, zinc, alkaline phosphatase, creatinine, and serum EDTA from a Becton Dickinson Sterile Sealed Tube II gel-containing tube collected before and after an EDTA-containing tube in 11 healthy volunteers using the closed blood collection system. The results showed no evidence to support the efficacy of the order of draw to prevent cross-contamination when closed blood collection is used [21]. 

Salvagno et al. [22] assessed 57 outpatients treated with oral anticoagulant and 58 healthy volunteers. In the outpatient group, a sodium citrate tube was collected before and after a serum tube; in the healthy volunteer group, an EDTA tube was collected before and after a serum tube. In both groups, no statistically significant difference in potassium, sodium, calcium, magnesium, or phosphorum emerged. The authors concluded the article affirming that this step of blood collection has a negligible importance and should no longer be used as a criterion in the evaluation of the performance of phlebotomists [22]. 

The observational and in vitro study carried out by Cadamuro et al. [23] tested the carryover of EDTA using distilled water as a substitute of blood and investigated copious blood values in 10 healthy volunteers using the open blood collection system (the filling order was Li-heparin tube, EDTA tube, Li-heparin tube). The authors simulated, at first, a blood collection of EDTA tube and subsequent non-additive tube; they then added increasing concentrations of EDTA in the heparin-containing tube, simulating the carryover of EDTA whole blood and pure EDTA. The results of the study showed minimal, and nonsignificant, EDTA contamination in the syringe collection experiment; in the in vitro part of the study, it emerged that a volume larger than 10 μL was necessary to alter the biochemical values. In conclusion, Cadamuro et al. asserted that the carryover during blood collection using a closed system is highly unlikely and even if it occurs, the volume needed to alter the test results is huge [23]. 

Sulaiman et al. [14] in their observational study recruited 10 healthy volunteers and investigated the blood values of EDTA, potassium, magnesium, calcium, zinc, alkaline phosphatase, and iron in a blood sample collected before and after an EDTA-containing tube using the Sarsted Safety Monovette System. After consulting the blood analysis values, the authors affirm that using this system of blood draw, the order of filling the tubes has no effect on serum biochemistry values [14]. 

The recent in vitro study of Keppel et al. [13] simulated the standardized phlebotomies to investigate the risk of carryover of citrate and heparin using distilled water as a substitute of blood. They also investigated the effect of the increasing level of citrate blood and citrate pure carryover in a lithium-containing tube. At the end of the study, the results suggested that during a standardized phlebotomy with a closed system, the risk of carryover seems negligible, but a small volume of additive is sufficient to cause a significant alteration of results when phlebotomy guidelines are not followed. To conclude, the authors asserted that cross-contamination should be considered when there is a suspicion of a spurious result [13]. 

In the latest study considered, Asif et al. [19] investigated all blood samples with potassium level ≥ 6 mmol/L over a period of 4 months, and at the end of the observation period 96 blood samples were found to be contaminated by EDTA. A total of 64 of 96 individuals responsible for the contaminated samples were identified and interviewed, and 52 of them remembered the blood collection system used. It came out that each one used the open phlebotomy method. In conclusion, the authors report that EDTA sample contamination is a trait of the open phlebotomy system, and the importance of following the order of draw in a closed system is not supported [19].

## 4. Discussion

This is the first systematic review that considers the order of draw and the blood collection system used for drawing. The order of draw is a highly debated issue, and the opinions of the authors were in conflict with each other. However, if more deeply investigated, this apparent discrepancy seems to be absent because the authors, although their results are different from each other, used different materials and techniques in their studies. In fact some authors conducted their studies using open blood collection system, while others used the closed blood collection system.

Some of the latest studies have focused not only on following the order or otherwise, but mostly on the real necessity to follow the order when using closed blood collection systems instead of the open system [14,19,20,21,23]. These studies suggest a new way to approach this controversial theme. In fact, they assert that the most important issue is not the order of sample tube blood filling but the system used. They discovered that using the closed blood collection system seems to avoid cross-contamination. This peculiar aspect appears to be the definitive turning point of this issue. Nevertheless, nowadays, the international recommendations for venipuncture indicate a strict and rigorous order of filling the blood collection tubes [4]. However, these indications are based on exiguous, anecdotal, and mostly past literature sources that were published more than 35 years ago using blood collection techniques and materials no longer in use [6,7]. Nevertheless, currently, the order of draw even when using the closed blood collection system continues to be recommended based on common sense rather than evidence [4,10,16]. Indeed, the international guidelines do not even mention these two different ways of approaching blood collection but only explain the open and closed blood collection systems in the blood-sampling systems chapter. Indeed, there are no hints about the possible differences in approaching blood tube filling in relation to the type of system used; however, both these methods are used in clinical practice worldwide [4,5]. One of the articles that affirm the importance of following the recommended order of draw underlines the statistically significant bias but at the same time asserts that the bias found does not have clinical consequences [25]. Probably, due to the fact that in some hospitals open and closed systems are both used, the guidelines tend to standardize the procedure. However, according to the most recent evidence, considering this finding might be an element of stream-lining of the whole procedure.

### Strengths and Limitations

The strengths of this review included compliance with the PRISMA guidelines for systematic reviews and the use of the CASP checklist to assess the methodological quality of the included studies [17,18]. In addition, potential bias was reduced through the involvement of more than two authors in the quality assessment, data extraction, and data analysis. The validity of the review is augmented by the recurrence of findings between studies. The strategy to involve more authors enabled the categorization of each finding reported in the studies without seeking to reinterpret the primary author’s findings. In addition, this approach guarantees the most objective evaluation of each study to ensure the highest credibility of the review.

Despite the rigor with which this review was conducted, some limitations need to be acknowledged. Firstly, although a comprehensive search on databases using the best keyword combinations was undertaken, publications not indexed in these databases could have been omitted. Other limitations include the studies’ heterogeneity, the huge odds of blood sample screened in the studies, the small number of recent sources in the literature, and the different materials used for blood collection. The difference in experience and training of the healthcare professionals (phlebotomists, nurses, or doctors) who performed the blood collection may represent a further limitation. Lastly, the present systematic review was not timely registered on PROSPERO.

## 5. Conclusions

According to the latest evidence, the focus of this problem actually seems to be the blood collection system used. The latest studies declare that the possibility of cross-contamination using the closed blood collection system with today’s materials and devices seems to be negligible even if the recommended order of filling tubes is not followed.

Indeed, in the case of blood collection using a closed system, the order of draw appears negligible. However, whenever blood collection is performed using an open system, the filling blood tube order would become essential to guarantee an exact biochemical result.

Because of the scarcity of recent studies in the literature about this issue, it is not possible to provide strong conclusions. Indeed, more empirical works are needed to define once and for all the effective importance of following the recommended order of draw.

## Figures and Tables

**Table 1:** Summary of selected studies.

Author (Year)	Journal	Aim	Type of Study	Samples	System of Draw	Main Findings	CASP
Cornes et al. (2008) [26]	Annals of Clinical Biochemistry	Measure EDTA in hyperkalemic samples to identify EDTA contamination.	Observational	117 inpatients and outpatients	Closed blood collection system	28 tubes were contaminated with EDTA. At low concentration, it is common, and it can only be confidently detected by measurement of serum EDTA. The order of draw is necessary to obtain correct biochemical results. EDTA contamination was not evident in blood samples collected by trained phlebotomists. The biochemical alterations are statistically and clinically significant.	10/12
Sharratt et al. (2009) [24]	International Journal of Clinical Practice	To audit the prevalence of EDTA contamination as a cause of hyperkalemia, hypocalcemia, hypomagnesemia, and hypozincemia.	Observational	12,895 patients	Closed blood collection system	31 tubes were contaminated with EDTA. Factious hyperkalemia, hypocalcemia, and hypomagnesemia and hypozincemia caused by EDTA contamination is relatively common, so the order of draw has importance. The spurious results are statistically and clinically significant.	11/12
Cornes et al. (2010) [15]	Clinical Laboratory	Measured EDTA in hyperkalemic (serum potassium ≥ 6.0 mmol/L) samples to determine the prevalence of EDTA sample contamination.	Observational	131,824 inpatients	Closed blood collection system	37 tubes were contaminated with EDTA. Hyperkalemia due to EDTA contamination is not uncommon and may remain undetected. The order of draw avoids spurious biochemical results. The results altered by EDTA are statistically and clinically significant.	9/12
Sulaiman et al. (2011) [14]	Journal of Clinical Pathology	To investigate whether incorrect order of draw of blood samples during phlebotomy causes in vitro potassium EDTA contamination of blood samples.	Observational	10 healthy volunteers	Closed blood collection system	0 tubes were contaminated with EDTA. The incorrect order of draw of blood samples does not result in EDTA sample contamination. In ideal phlebotomy conditions, the order of draw has no effect on serum biochemical results.	11/12
Fukugawa et al. (2012) [25]	American Journal of Clinical Pathology	To investigate the effect of clot activators carried over from the serum in major coagulation tests during phlebotomy.	Observational and in vitro	100 (75 healthy volunteers and 25 patients)	Closed blood collection system	Using standard phlebotomy sequence, it may be accepFIGUREto collect the coagulation after the serum tube. The order of draw has statistically but not clinically significant importance.	10/12
Cornes et al. (2012) [21]	British Journal of Biomedical Science	To investigate whether reversed order of draw of blood causes in vitro potassium EDTA contamination.	Observational	11 healthy volunteers	Closed blood collection system	0 tubes were contaminated with EDTA. Reversed order of draw of blood samples does not cause potassium EDTA sample contamination, irrespective of the type of closed blood collection system used. The draw was collected by the same experienced phlebotomist.	10/12
Salvagno et al. (2013) [22]	Clinical Chemistry and Laboratory Medicine	To establish whether or not following a specific order of draw is still reasonable or analytically and clinically justified.	Observational	115 (57 outpatients and 58 healthy volunteers)	Closed blood collection system	0 tubes were contaminated with EDTA. The order of draw has a negligible importance even when the internal needle of the holder gets in contact with the blood/additive mixture. It should be no longer considered a quality criterion in the evaluation of the performance of phlebotomists.	11/12
Indevuyst et al. (2015) [20]	International Journal of Laboratory Hematology	To evaluate the effect of the order of draw on the PT/INR and aPTT.	Observational	193 patients	Closed blood collection system	The order of draw has no significant influence on PT/INR but biases the aPTT without clinical consequence. The venipunctures were performed by experienced phlebotomists. The order of draw for modern vacuum tube collection systems is indeed “much ado about nothing.”	10/12
Cadamuro et al. (2015) [23]	Clinical Chemistry and Laboratory Medicine	To investigate the principle of EDTA carryover during venipuncture using the closed vacuum system and EDTA contamination in vitro by simulating specimen collection.	Observational and in vitro	10 healthy volunteers	Open bloodcollection system	0 tubes were contaminated with EDTA. The carryover during blood collection using a closed system is highly unlikely and, even if it occurs, the volume needed to alter the test results is huge. The order of draw, adhering to the current recommendations in blood collection, could not be followed.	10/12
Keppel et al. (2019) [13]	Clinical Chemistry and Laboratory Medicine	To assess effects of potential carryover of citrate and heparin additives during a standard phlebotomy procedure.	In vitro	10 tubes	Closed blood collection system	Sample contamination with additives from other tubes can occur only if guidelines on blood collection are not strictly followed or an open blood-sampling system is used. The effect of order of draw using closed blood collection system seems to be negligible.	11/12
Asif et al. (2019) [19]	Annals of Clinical Biochemistry	To identify the causes of EDTA contaminated samples in routine clinical practice.	Observational	96 patients	Open blood collection system	EDTA sample contamination is a trait of the open phlebotomy system. The guidelines should emphasize the use of closed blood collection systems and underline the need to follow the order of draw only when using open phlebotomy methods.	11/12

CASP: Critical Appraisal Skill Program; EDTA: ethylenediaminetetraacetic acid; PT: prothrombin time; aPTT: activated partial thromboplastin time; INR: international normalized ratio.

## Data Availability

Not applicable.
